# A single-institution retrospective analysis of pathologically determined malignant transformation in *IDH* mutant glioma patients

**DOI:** 10.1093/noajnl/vdad036

**Published:** 2023-04-11

**Authors:** Vicki Liu, Ethan A Wetzel, Blaine S C Eldred, Serendipity Zapanta Rinonos, Terry J Prins, Negar Khanlou, Linda M Liau, Robert Chong, Phioanh L Nghiemphu, Timothy F Cloughesy, Benjamin M Ellingson, Albert Lai

**Affiliations:** Department of Neurology, David Geffen School of Medicine, University of California–Los Angeles, Los Angeles, California, USA; Department of Neurology, David Geffen School of Medicine, University of California–Los Angeles, Los Angeles, California, USA; Department of Neurology, David Geffen School of Medicine, University of California–Los Angeles, Los Angeles, California, USA; Department of Neurology, David Geffen School of Medicine, University of California–Los Angeles, Los Angeles, California, USA; Department of Neurology, David Geffen School of Medicine, University of California–Los Angeles, Los Angeles, California, USA; Department of Pathology and Laboratory Medicine, David Geffen School of Medicine, University of California–Los Angeles, Los Angeles, California, USA; Department of Neurosurgery, David Geffen School of Medicine, University of California–Los Angeles, Los Angeles, California, USA; Department of Neurology, David Geffen School of Medicine, University of California–Los Angeles, Los Angeles, California, USA; Department of Neurology, David Geffen School of Medicine, University of California–Los Angeles, Los Angeles, California, USA; Department of Neurology, David Geffen School of Medicine, University of California–Los Angeles, Los Angeles, California, USA; Department of Radiological Sciences, David Geffen School of Medicine, University of California–Los Angeles, Los Angeles, California, USA; Department of Neurology, David Geffen School of Medicine, University of California–Los Angeles, Los Angeles, California, USA

**Keywords:** glioma, IDH1/2, malignant transformation, progression, tumor grade

## Abstract

**Background:**

Lower-grade *IDH* mutant glioma patients frequently undergo malignant transformation (MT), with apparent worse prognosis. Many studies examine MT in mixed *IDH* status cohorts and define MT using imaging, not histopathology. Our study examines the timing, predictors, and prognostic implications of pathologically determined MT in a large, exclusively *IDH* mutant cohort.

**Methods:**

We identified 193 *IDH* mutant lower-grade glioma patients at UCLA who received multiple surgeries. We examined the outcomes of pathologically determined MT patients.

**Results:**

Time to MT is longer in grade 2 oligodendroglioma (G2 Oligo) than in grade 2 astrocytoma (G2 Astro) (HR = 0.46, *P* = .0007). The grade 3 astrocytoma (G3 Astro) to grade 4 astrocytoma (G4 Astro) interval is shorter in stepwise MT (G2 to G3 to G4 Astro) patients than in initial G3 Astro patients (*P* = .03). Novel contrast enhancement had 65% positive predictivity, 67% negative predictivity, 75% sensitivity, and 55% specificity in indicating pathologically defined MT. In G2 Astro, initial gross total resection delayed MT (HR = 0.50, *P* = .02) and predicted better overall survival (OS) (HR = 0.34, *P* = .009). In G2 Oligo, spontaneous MT occurred earlier than treated MT (HR = 11.43, *P* = .0002), but treatment did not predict improved OS (*P* = .8). MT patients (*n* = 126) exhibited worse OS than non-MT patients (*n* = 67) in All (HR = 2.54, *P* = .0009) and G2 Astro (HR = 4.26, *P* = .02).

**Conclusion:**

Our study expands the understanding of MT to improve *IDH* mutant lower-grade glioma management.

**Key Points**

- Malignant transformation (MT) is detrimental to overall survival (OS) in G2 Astro, but not in G2 Oligo or G3 Astro.
- Initial gross total resection prolongs MT-free survival and OS in G2 Astro.
- Spontaneous MT occurs earlier but shows better OS than treated MT, though this may reflect selection bias.

**Importance of the Study**

Malignant transformation (MT) is poorly understood in *IDH* mutant lower-grade gliomas, as most existing retrospective studies include patients of mixed *IDH* status. Furthermore, novel MRI contrast enhancement is often relied upon as an indicator of tumor progression after initial surgery, though this method is unable to distinguish between MT and nontransforming progression. Our study examines pathologically determined MT in a large cohort of 193 exclusively *IDH* mutant lower-grade glioma patients. We found that 65% of patients with additional surgeries experienced MT, which correlated with worse survival in low-grade astrocytomas, but not oligodendrogliomas. Additionally, we describe the effects of extent of resection and treatment on time to MT and overall survival in MT patients. Overall, this study expands our understanding of MT, revealing its implications and predictors across glioma subtypes. This will inform clinical strategies to anticipate and reduce MT risk in *IDH* mutant lower-grade glioma patients.

Gliomas, the most common primary brain cancer in adults, are diagnosed based on the WHO Classification of Tumors of the Central Nervous System (CNS). Under 2021 WHO standards, *IDH* mutant gliomas are graded based on histology, immunohistochemistry, and molecular parameters, where a higher grade indicates more aggressive disease and worse prognosis.^1,2^ Astrocytomas, molecularly defined by intact 1p19q chromosomal arms, can be assigned to grade 2 astrocytoma (G2 Astro), grade 3 astrocytoma (G3 Astro), or grade 4 astrocytoma (G4 Astro), whereas oligodendrogliomas, molecularly defined by 1p19q codeletion, can be assigned to grade 2 oligodendroglioma (G2 Oligo) or grade 3 oligodendroglioma (G3 Oligo). Despite receiving an initial diagnosis of lower-grade glioma, a subset of G2 Astro, G3 Astro, and G2 Oligo patients experience disease progression to a higher grade through a poorly understood process known as malignant transformation (MT).

*Isocitrate dehydrogenase* (*IDH*) mutations are present in 68% of lower-grade gliomas and play a key role in informing the treatment and prognosis of glioma patients.^3^ MT affects both *IDH* mutant and wild-type patients, at an estimated incidence of 45% in oligodendrogliomas and 74% of astrocytomas.^4^ Prior studies on MT were overwhelmingly conducted in patient cohorts of mixed *IDH* status. However, *IDH* mutant and wild-type gliomas behave differently and MT occurs at higher rates in *IDH* mutant gliomas,^5^ despite their less aggressive clinical course.^2^ Attempts to identify predictive markers have associated increased MT risk with older age,^6,7^ male sex,^6–8^ larger tumor volume,^6,8^ and multiple tumor locations.^7^ In terms of modifiable clinical factors, lower extent of resection^9–11^ and temozolomide (TMZ) treatment^8,12,13^ have been implicated as risk factors for MT. Recently, magnetic resonance imaging (MRI) parameters^14^ and genomic features^15^ have also shown promise in detecting and understanding MT. For instance, one clinical evaluation suggests that MT occurs local to the initial tumor and within T2-weighted hyperintense regions.^16^

Notably, many prior studies defined MT based on changes in contrast enhancement in the absence of pathology. However, there are no broadly accepted guidelines for differentiating between MT and progression within the same grade using radiographic features. A recent study assessing the ability of new MRI enhancement to serve as a surrogate of pathologic grade found that enhancement failed to identify MT in over 20% of cases.^17^ Given the increased MT risk in *IDH* mutant patients, lack of studies examining MT in exclusively *IDH* mutant cohorts, and the imprecise definition of MT in other studies, we sought to investigate the incidence rates, survival implications, and clinical indications of pathologically determined MT in 193 lower-grade *IDH* mutant glioma (G2 Astro, G3 Astro, G2 Oligo) patients.

## Materials and Methods

### Patient Cohort

In this UCLA Institutional Review Board-approved study, informed patient consent was obtained prior to the collection and analysis of data. We complied with the 2016 WHO Classification of Tumors of the CNS, as many patient records lacked key genetic testing necessary for the updated 2021 WHO criteria (e.g. *CDKN2A* loss). Initial and subsequent diagnoses were determined by direct review of reports from board-certified pathologists. For obsolete categories, namely mixed oligoastrocytomas, histopathological evaluation and molecular testing were used to update diagnoses. Although our patient cohort dates back to 1986, all patients had confirmed 1p19q codeletion status based on clinical testing, typically by fluorescence in situ hybridization (FISH).

We retrospectively identified 454 *IDH* mutant lower-grade glioma patients, seen at UCLA between 1986 and 2021. This included 152, 147, and 155 patients initially diagnosed with grade 2 astrocytoma (G2 Astro), grade 3 astrocytoma (G3 Astro), and grade 2 oligodendroglioma (G2 Oligo), respectively. Of these, 195 patients received at least one additional surgery after initial surgery and were thus included for stratification into MT versus non-MT groups.

### Pathological Definition of MT

For each patient, we reviewed all available pathology reports corresponding to tissue resected at initial and subsequent surgeries. Among the 195 patients with additional surgeries, 176 (90%) patients had pathology reviewed at UCLA and 19 (10%) had pathology reviewed at other institutions only, but later received treatment or consultation at UCLA. Patients were placed in the MT group if they experienced an increase in tumor grade between initial surgery and any subsequent surgery, as determined by board-certified pathologists. Patients were placed in the non-MT group if their tumors remained the same grade at all subsequent surgeries. Based on these definitions, we identified 128 patients with MT and 67 patients without MT.

In the non-MT group, we identified 4 instances of pseudoprogression (PsP), which was pathologically defined based on a Ki67 value below 1% and the absence of neoplastic features (hypercellularity, cellular or nuclear atypia, mitoses). Two of these patients had later surgeries revealing nontransforming progression, whereas 2 had PsP only, with no subsequent surgeries revealing true tumor tissue. Because MT did not occur, these PsP-only patients (1 G2 Astro and 1 G3 Astro) were included with nontransforming progression patients as part of the non-MT group in our analyses.

In the MT group, we sought to minimize the possibility of false calls of MT, generated by inaccurate grading at initial diagnosis. Because it is unlikely for a tumor to transform to a higher grade in an extremely short timeframe, patients with an interval between surgeries of less than 3 months were not placed in the MT group.^4^ In accordance to this, we identified and excluded 1 initial G2 Astro patient and 1 initial G2 Oligo patient who received repeat resections 0.6 and 2.2 months after their initial surgeries.

In our resulting cohort of 193 *IDH* mutant lower-grade glioma patients who received additional surgeries, there were 85 patients initially diagnosed with G2 Astro, 47 with G3 Astro, and 61 with G2 Oligo. 126 (65%) had MT, including 64 (75%) G2 Astros, 23 (49%) G3 Astros, and 39 (64%) G2 Oligos. Twenty-six initial G2 Astro patients experienced MT to G3 Astro, 28 experienced MT to G4 Astro, and 10 experienced MT to G3 followed by MT to G4 Astro (“stepwise” MT). Relevant clinical information, including patient demographics, survival data, and magnetic resonance imaging, was recorded based on available electronic medical records. See Table 1 for cohort characteristics, Supplementary Figure S1 for a flowchart of the patient selection process, and Supplementary Table S1 for the surgeries at which MT was observed.

**Table 1.** Patient Cohort

Characteristics	Patients	MT (%)	Non-MT (%)
All	193	126 (65)	67 (35)
Median age at diagnosis	33.2	32.9	33.7
Sex			
Male	118	76 (64)	42 (36)
Female	75	50 (67)	25 (33)
Initial diagnosis			
G2 Astro	85	64 (75)	21 (25)
G3 Astro	47	23 (49)	24 (51)
G2 Oligo	61	39 (64)	22 (36)
Initial extent of resection			
Biopsy only	23	17 (74)	6 (26)
STR	90	54 (60)	36 (40)
GTR	74	50 (68)	24 (32)
Unknown	6	5 (83)	1 (17)
Treatment prior to additional surgery			
Radiation only	37	34 (92)	3 (8)
Chemotherapy only	22	17 (77)	5 (23)
Chemotherapy and radiation	69	41 (59)	28 (41)
No chemotherapy or radiation	65	34 (52)	31 (48)
*MGMT* status			
Methylated	67	48 (72)	19 (28)
Unmethylated	45	28 (62)	17 (38)
Unknown	81	50 (62)	31 (38)

Demographic characteristics of 193 *IDH* mutant lower-grade glioma patients who received additional surgeries in UCLA cohort. G2 Astro, grade 2 astrocytoma; G3 Astro, grade 3 astrocytoma; G2 Oligo, grade 2 oligodendroglioma; STR, subtotal resection; GTR, gross total resection; *MGMT*, *O6-methylguanine-DNA- methyltransferase*; MT, malignant transformation.

### Triggers for Surgery

For each patient, we documented the primary reason for all additional surgeries based on patient records from board-certified neuro-oncologists and neurosurgeons. We found 3 categories of surgical triggers, including CEnew, T2 change, and repeat craniotomy. One hundred and eighteen patients (51 G2 Astro, 37 G3 Astro, 30 G2 Oligo at initial diagnosis) received second surgeries due to CEnew, which was defined as the appearance of novel contrast enhancement following initial surgery on T1-weighted MRI scans with gadolinium-based contrast. The remaining patients did not exhibit CEnew but received surgery for other reasons. Fifty-nine patients (27 G2 Astro, 5 G3 Astro, 27 G2 Oligo at initial diagnosis) received second surgeries due to a T2 change, or slow expansion of the tumor seen on T2-weighted MRI scans. Sixteen patients (7 G2 Astro, 5 G3 Astro, 4 G2 Oligo at initial diagnosis) received repeat craniotomies for further debulking to improve extent of resection, in the absence of evidence for progression. The median time interval between a patient’s surgical trigger and subsequent second surgery was 0.95 months. See Supplementary Table S2 for a summary of triggers. We compared MT versus non-MT outcome at second surgery following each of these triggers using the Fisher’s exact test, to evaluate their potential to predict MT. Furthermore, we calculated the positive predictive, negative predictive, specificity, and sensitivity values of CEnew as an indicator of MT.

### Statistical Analyses

Kaplan–Meier curves were generated to compare survival between groups. Overall survival (OS) was defined as the time between a patient’s initial surgery and date of death or last follow-up. For MT patients, residual overall survival (resOS) was defined as the time between the surgery at which MT was first pathologically discovered and the date of death or censoring. Because MT was defined based on pathology only, time to MT was defined as the time between initial surgery and MT surgery, irrespective of instances of radiographic progression.

Multivariate analyses using the Cox proportional hazards model were also performed to evaluate the predictive potential of various clinical and radiographic markers. *P* values of .05 or below were considered statistically significant. The following covariates were included: age at initial surgery, sex, Karnofsky Performance Scale (KPS), initial extent of resection, initial glioma subtype, treatment with chemotherapy and/or radiation (Tables 2 and 4, Supplementary Table S9 only), and MT (Supplementary Table S7 only). For the 10 patients that experienced stepwise MT from G2 Astro to G3 then G4 Astro, time to MT was defined as the interval between initial G2 Astro surgery and the surgery yielding G4 Astro pathology, as this reflected the entire course of MT. Six patients (5 MT and 1 non-MT) with unknown initial extents of resection were not included in our multivariate analyses.

**Table 2.** Multivariate of Time to MT

Variable (Time to MT)	All (*n* = 121)			G2 Astro (*n* = 62)			G3 Astro (*n* = 22)			G2 Oligo (*n* = 37)		
	HR	*P* value	95% CI	HR	*P* value	95% CI	HR	*P* value	95% CI	HR	*P* value	95% CI
Age at initial surgery	1.03	.004*	[1.01, 1.05]	1.05	.001*	[1.02, 1.08]	0.99	.9	[0.94, 1.06]	1.03	.2	[0.99, 1.07]
Male (ref. female)	0.83	.4	[0.55, 1.25]	0.55	.06	[0.30, 1.01]	1.87	.3	[0.64, 5.45]	0.69	.3	[0.33, 1.41]
KPS ≤ 70	0.51	.07	[0.25, 1.06]	0.15	.006*	[0.04, 0.59]	0.90	.9	[0.13, 6.14]	1.01	1	[0.30, 3.44]
GTR (ref. STR/biopsy)	0.79	.3	[0.53, 1.18]	0.50	.02*	[0.27, 0.91]	0.66	.4	[0.23, 1.87]	1.35	.5	[0.53, 3.41]
Initial G3 Astro (ref. G2 Astro)	1.03	.9	[0.62, 1.73]	—	—	—	—	—	—	—	—	—
Initial G2 Oligo (ref. G2 Astro)	0.46	.0007*	[0.30, 0.72]	—	—	—	—	—	—	—	—	—
No treatment (ref. chemotherapy and/or radiation)	2.31	.0003*	[1.47, 3.65]	1.54	.1	[0.87, 2.72]	2.48	.4	[0.25, 24.57]	11.43	.0002*	[3.11, 42.05]
Events (%)	121 (100)			62 (100)			22 (100)			37 (100)		

Multivariate analysis of time to pathologically defined MT in MT patients with known EOR, stratified by initial diagnoses. G2 Astro, grade 2 astrocytoma; G3 Astro, grade 3 astrocytoma; GTR, gross total resection; KPS, Karnofsky Performance Scale; MT, malignant transformation; STR, subtotal resection.

*A statistically significant association (*P* < .05 by Cox regression analysis).

**Table 4.** Multivariate of OS in Malignant Transformation Patients

Variable (OS)	All (*n* = 121)			G2 Astro (*n* = 62)			G3 Astro (*n* = 22)			G2 Oligo (n = 37)		
	HR	*P* value	95% CI	HR	*P* value	95% CI	HR	*P* value	95% CI	HR	*P* value	95% CI
Age at initial surgery	1.01	.4	[0.98, 1.04]	1.02	.2	[0.99, 1.06]	1.03	.3	[0.97, 1.10]	1.03	.3	[0.97, 1.11]
Male (ref. female)	0.79	.4	[0.47, 1.35]	0.45	.03*	[0.22, 0.92]	2.96	.1	[0.80, 10.99]	0.63	.4	[0.22, 1.80]
KPS ≤ 70	0.92	.8	[0.40, 2.08]	0.10	.008*	[0.02, 0.55]	5.56	.06	[0.87, 31.90]	4.04	.05*	[1.01, 16.18]
GTR (ref. STR/biopsy)	0.72	.2	[0.43, 1.21]	0.34	.009*	[0.15, 0.76]	0.87	.8	[0.26, 2.94]	1.20	.8	[0.36, 3.95]
Initial G3 Astro (ref. G2 Astro)	1.41	.3	[0.76, 2.61]	—	—	—	—	—	—	—	—	—
Initial G2 Oligo (ref. G2 Astro)	0.38	.002*	[0.21, 0.70]	—	—	—	—	—	—	—	—	—
No treatment (ref. chemotherapy and/or radiation)	0.51	.05*	[0.26, 1.00]	0.20	.001*	[0.08, 0.53]	0.36	.4	[0.03, 4.31]	1.21	.8	[0.32, 4.62]
Events (%)	70 (58)			37 (60)			16 (73)			17 (46)		

Multivariate analysis of OS in MT patients with known EOR, stratified by initial diagnoses. G2 Astro, grade 2 astrocytoma; G3 Astro, grade 3 astrocytoma; GTR, gross total resection; KPS, Karnofsky Performance Scale; OS, overall survival; MT, malignant transformation; STR, subtotal resection.

*A statistically significant association (*P* < .05 by Cox regression analysis).

## Results

### Time to MT Is Longest for G2 Oligo

To investigate the time to MT in *IDH* mutant glioma patients, we first identified 193 patients with initial G2 Astro, G3 Astro, and G2 Oligo that had additional surgeries. We found that 126 (65%) experienced surgically determined MT, whereas 67 (35%) had no evidence of MT on repeat pathology. We found MT occurring in 75% of G2 Astros (64/85), 49% of G3 Astros (23/47), and 64% of G2 Oligos (39/61) (Table 1). Of the 64 G2 Astro patients with MT, 26 transformed to G3 Astro only, 28 transformed directly to G4 Astro, and 10 transformed to G3 Astro then G4 Astro, which we termed “stepwise” MT (Supplementary Figure S2). For the patients without stepwise MT, the median times to MT (months) were as follows: 47.5 for G2 to G3 Astro, 46.9 for G2 to G4 Astro, and 47.3 for G3 to G4 Astro. For the 10 patients with stepwise MT, a median of 32.9 months elapsed from the initial G2 Astro surgery to the G3 Astro surgery, followed by 12.5 months from the G3 Astro surgery to the G4 Astro surgery. The time to MT from G2 to G4 Astro was similar between patients with stepwise MT and patients who did not receive an intermediate diagnosis of G3 Astro (Supplementary Figure S3C, Supplementary Table S3). However, compared with initial G3 Astro patients, these stepwise patients exhibited a much shorter time to MT between their G3 and G4 Astro diagnoses (*P* = .03) (Supplementary Figure S3B).

Compared with astrocytoma patients, G2 Oligo patients had a longer time to MT, with a median G2 to G3 Oligo interval of 82.4 months. Multivariate analysis confirmed that the G2 Oligo subtype is associated with a delayed time to MT compared with the G2 Astro subtype (HR = 0.46, *P* = .0007) (Table 2). Overall, these results indicate that the interval between initial surgery and MT is longer in G2 Oligo than in G2 Astro, and the time to MT from G3 to G4 Astro is shorter in stepwise MT G2 Astro patients than in initial G3 Astro patients.

### CEnew as a Trigger for Surgery Is an Imperfect Indicator of MT

Because the appearance of novel postoperative contrast enhancement (CEnew) has commonly been accepted as an imaging marker of MT,^6–8,11,18^ we identified the “trigger” for additional surgeries and analyzed the accuracy of CEnew as a trigger in identifying pathologically confirmed MT. Focusing on second surgeries only, a total of 118 patients received second surgeries due to appearance of CEnew. Seventy-seven of these 118 (65%) patients experienced MT at second surgery, including 39/51 (76%) G2 Astros, 20/37 (54%) G3 Astros, and 18/30 (60%) G2 Oligos. The remaining 75 patients received second surgeries due to other triggers, including T2 changes (*n* = 59 total, 27 G2 Astros, 5 G3 Astros, 27 G2 Oligos) and repeat or redo craniotomies (*n* = 16 total, 7 G2 Astros, 5 G3 Astros, 4 G2 Oligos). Twenty-five of these 75 (33%) patients without CEnew experienced MT at second surgery, which includes 16/34 (47%) G2 Astros, 1/10 (10%) G3 Astros, and 8/31 (26%) G2 Oligos. By Fisher’s exact test, CEnew preceding surgery was a better predictor of MT than other surgical triggers in All (*P* < .0001), G2 Astros (*P* = .01), G3 Astros (*P* = .02), and G2 Oligos (*P* = .01) (Table 3).

**Table 3.** Predictive Value of CEnew for MT

Initial Diagnosis	Trigger for Surgery	Total	MT (%)	No MT (%)
G2 Astro	CEnew	51	39 (76)*	12 (24)
	Other	34	16 (47)	18 (53)
G3 Astro	CEnew	37	20 (54)*	17 (46)
	Other	10	1 (10)	9 (90)
G2 Oligo	CEnew	30	18 (60)*	12 (40)
	Other	31	8 (26)	23 (74)
All	CEnew	118	77 (65)*	41 (35)
	Other	75	25 (33)	50 (67)

Table evaluating the predictive potential of CEnew as a trigger for surgery with regards to MT outcome in all, G2 Astro, G3 Astro, and G2 Oligo patients. Trigger and corresponding outcome for second surgery are shown. G2 Astro, grade 2 astrocytoma; G3 Astro, grade 3 astrocytoma; G2 Oligo, grade 2 oligodendroglioma; MT, malignant transformation.

*A statistically significant association between MT and CEnew (*P* < .05 by Fisher’s exact test).

Using this data, we calculated positive predictivity, negative predictivity, sensitivity, and specificity values of CEnew as a marker of MT. We found that CEnew had an overall positive predictivity of 65%, negative predictivity of 67%, sensitivity of 75%, and specificity of 55% in all patients (Supplementary Table S4). In individual diagnoses, the positive predictive values were 76%, 54%, and 60%, and the negative predictive values were 53%, 90%, and 74% in G2 Astro, G3 Astro, and G2 Oligo patients, respectively. The sensitivity values were 71%, 95%, and 69%, and the specificity values were 60%, 35%, and 66%, respectively. Although MT was observed at a significantly higher frequency (65%) in surgeries triggered by CEnew, MT was also discovered in 33% of surgeries triggered by other reasons. Overall, these results demonstrate that the accuracy of CEnew as a marker of MT is limited.

### Initial Gross Total Resection Delays MT in G2 Astro Patients

In addition to glioma subtype, we sought to identify other clinical factors that could predict time to MT. Kaplan–Meier analyses showed that G2 Astro MT patients who received initial gross total resection (GTR) had a longer time to MT than those who received initial subtotal resection (STR)/biopsy (*P* = .03) (Supplementary Figure S4B). Multivariate analysis further demonstrated that initial GTR was associated with a longer time to MT compared to STR/biopsy in G2 Astro patients (HR = 0.50, *P* = .02) (Table 2). This association was not observed in the All group (HR = 0.79, *P* = .3) or in any other individual subtype (G3 Astro: HR = 0.66, *P* = .4; G2 Oligo: HR = 1.35, *P* = .5) (Table 2). Taken together, GTR delays MT in patients with initial G2 Astro, but not G3 Astro or G2 Oligo.

### Spontaneous MT Occurs Earlier Than Treatment-Associated MT in G2 Oligo Patients

We also explored the impact of prior treatment on time to MT. Of the 126 MT patients, 34 (27%) received radiation alone, 17 (13%) received chemotherapy alone, in the form of temozolomide and/or procarbazine hydrochloride, lomustine, vincristine sulfate (TMZ/PCV), and 41 (33%) MT patients received both chemotherapy and radiation. Interestingly, 34 (27%) MT patients did not receive any standard-of-care treatment prior to MT, which we designated as “spontaneous” MT. Thirty spontaneous MT patients were under observation only prior to MT, while the remaining 4 (3 G2 Astro and 1 G3 Astro) were treated with small-molecule *IDH* inhibitors. Kaplan–Meier analyses demonstrated that spontaneous MT patients have an earlier time to MT compared with treated MT patients (*P* = .01) (Supplementary Figure S5C). Multivariate analyses also showed that compared with chemotherapy and/or radiation, the absence of treatment (spontaneous MT) was associated with an earlier time to MT in All patients (HR = 2.31, *P* = .0003) (Table 2). In individual subtypes, this difference was observed in G2 Oligo (HR = 11.43, *P* = .0002), but not in G2 Astro (HR = 1.54, *P* = .1) or G3 Astro (HR = 2.48, *P* = .4) (Table 2).

We sought to evaluate the 34 spontaneous MT patients in closer detail to examine the natural history of MT. This included 12 G2 to G3 Astro, 1 G2 to G3 to G4 Astro, 5 G2 to G4 Astro, 2 G3 to G4 Astro, and 14 G2 to G3 Oligo patients. In multivariate analysis of spontaneous MT patients, we found that older age at initial surgery was associated with accelerated time to MT (HR = 1.07, *P* = .001), whereas GTR delayed spontaneous MT (HR = 0.35, *P* = .02) (Supplementary Table S5). Although we found the G2 Oligo subtype to be associated with later MT in our analysis of All MT patients, interestingly, this was not seen in spontaneous MT patients (HR = 1.48, *P* = .4) (Supplementary Table S5). We also found that CEnew was less likely to precede spontaneous MT compared with treated MT (Fisher’s exact test *P* = .02) (Supplementary Table S6). Overall, these results indicate that spontaneous MT occurs earlier than treated MT in G2 Oligo patients. Spontaneous MT presents less frequently with CEnew and may be delayed by initial GTR.

### MT Is Detrimental to Survival in G2 Astro Patients, but not G2 Oligo or G3 Astro Patients

**Figure 1.**
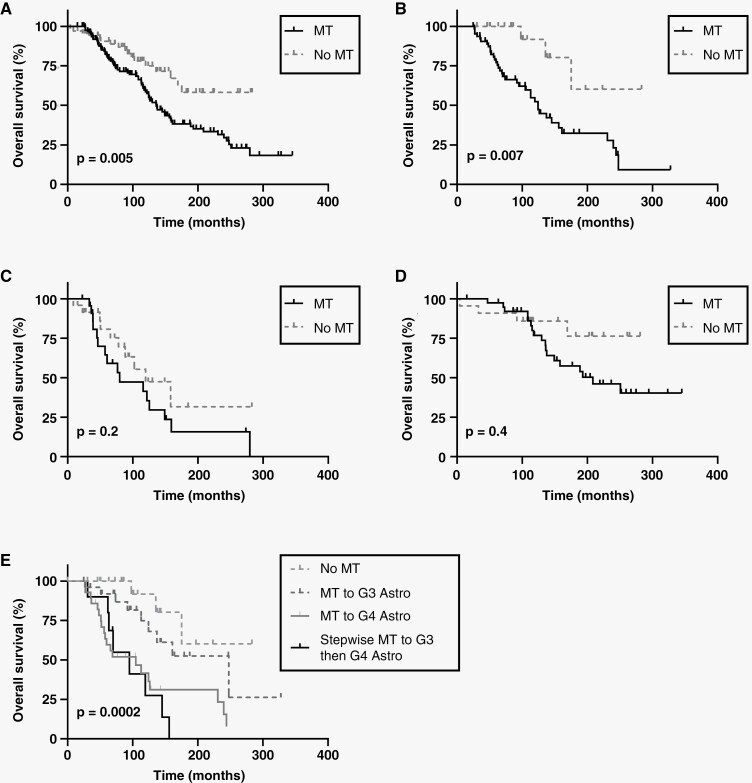
Prognostic impact of MT on OS. (A–D) Univariate Kaplan–Meier analysis comparing OS of MT versus non-MT patients for (A) all relevant glioma subtypes (*P* = .005), (B) initial G2 Astro (*P* = .007), (C) initial G3 Astro (*P* = .2), and (D) initial G2 Oligo (*P* = .4). (E) Comparison of OS of initial G2 Astro patients across outcome groups (*P* = .0002). G2 Astro, grade 2 astrocytoma; G3 Astro, grade 3 astrocytoma; OS, overall survival; MT, malignant transformation.

To evaluate the implications of MT on survival, we performed Kaplan–Meier and Cox multivariate analyses to compare OS between pathological MT and non-MT groups. The median OS for all patients who had MT (*n* = 126) was 136.0 months, whereas the non-MT patients (*n* = 67) did not reach a median OS (*P* = .005) (Figure 1A). In concordance with univariate results, multivariate analysis of all patients with known extent of resection (*n* = 187) showed that MT was a significant predictor of worse OS (HR = 2.54, *P* = .0009) (Supplementary Table S7).

We further examined whether the prognostic effects of MT differed based on glioma subtype and found that the negative impact of MT on OS was exclusive to G2 Astro. For initial G2 Astro, MT patients (*n* = 64) had worse OS, with a median of 124.2 months, compared with non-MT patients (*n* = 21) who did not reach a median OS (*P* = .007) (Figure 1B). This was also seen in multivariate analysis (HR = 4.26, *P* = .02) (Supplementary Table S7). Further univariate analysis revealed that initial G2 Astro patients that undergo stepwise MT (*n* = 10, median OS = 95.0 months) or undergo MT to G4 Astro directly (*n* = 28, median OS = 104.7 months) perform much worse than those that transform to G3 Astro only (n = 26, median OS = 247.6) (*P* = .0002) (Figure 1E). G2 to G4 Astro patients had similar OS, regardless of whether they received an intermediate diagnosis of G3 Astro (stepwise MT) or not (Supplementary Table S3). We did not find any differences in characteristics between patients with stepwise MT and patients with MT from G2 to G3 Astro only (Supplementary Table S8).

G3 Astro patients who undergo MT (*n* = 23) had a median OS of 80.0 months versus 119.6 months for non-MT G3 Astro patients (*n* = 24), although this difference was not significant (*P* = .2) (Figure 1C). Likewise, for initial G2 Oligo patients, MT (*n* = 39) was not associated with decreased OS, corresponding to a median OS of 208.5 months compared with non-MT (n = 22), which led to no median OS (*P* = .4) (Figure 1D). This was also seen in multivariate analysis, where MT was not associated with worse OS in G3 Astro (HR = 1.54, *P* = .4) or G2 Oligo (HR = 2.62, *P* = .1) (Supplementary Table S7).

Although MT predicts worse OS in our combined cohort of all lower-grade patients, stratification by glioma subtype reveals that MT from G2 Astro to G3 or G4 Astro correlates with worse OS, indicating that MT is detrimental to clinical outcome in initial G2 Astro patients, but not in initial G3 Astro or G2 Oligo patients.

### Initial Gross Total Resection and Spontaneous MT Is Associated With Improved OS in G2 Astro Patients

We then sought to understand factors influencing survival in MT patients. Our Kaplan–Meier analyses indicate that in addition to delaying MT, GTR correlates with better OS in G2 Astro MT patients (*P* = .03) (Supplementary Figure S4B). Multivariate analysis supported that GTR at initial surgery predicts better OS compared to STR/biopsy in G2 Astro MT patients (HR = 0.34, *P* = .009) (Table 4). As with time to MT, no significant correlations between extent of resection and OS were observed among MT patients in All (HR = 0.72, *P* = .2) or other subtypes (G3 Astro: HR = 0.87, *P* = .8; G2 Oligo: HR = 1.20, *P* = .8) (Table 4).

**Figure 2.**
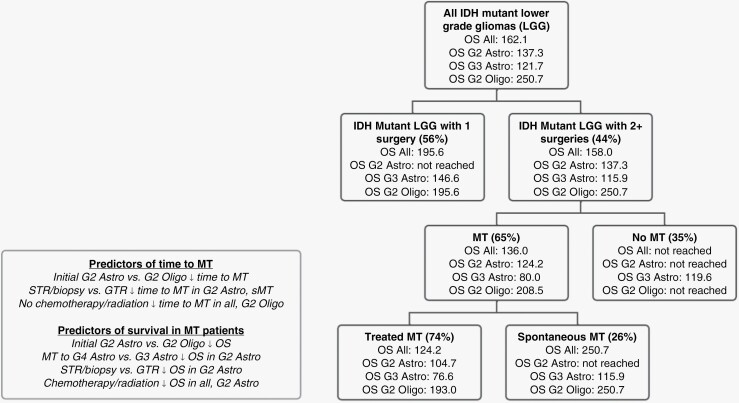
Survival implications and predictors of MT. Flowchart depicting the median OS (months) of *IDH* mutant lower-grade glioma patients, separated by initial surgery only versus additional surgeries, MT versus non-MT, and treated MT versus spontaneous MT. Predictors of time to MT and OS in MT patients are also shown. OS, overall survival; MT, malignant transformation.

Kaplan–Meier analyses indicate that spontaneous MT correlates with better OS (*P* = .01) and resOS (*P* < .0001), despite an earlier time to MT (*P* = .01) compared to treatment-associated MT (Supplementary Figure S5). Although our multivariate results also indicated that standard-of-care treatment is effective at prolonging MT-free survival in G2 Oligo, we did not find any OS benefit associated with treatment when comparing spontaneous vs. treated G2 Oligo MT patients (HR = 1.21, *P* = .8) (Table 4). Likewise, treatment did not have prognostic value in G3 Astro MT patients (HR = 0.36, *P* = .4). Instead, spontaneous MT correlated with better OS in All (HR = 0.51, *P* = .05) and G2 Astro MT patients (HR = 0.20, *P* = .001) (Table 4). Multivariate analyses also show that spontaneous MT predicts better residual OS (resOS) in All (HR = 0.20, *P* < .0001) and G2 Astro (HR = 0.18, *P* = .0007) (Supplementary Table S9). Overall, our results suggest that initial GTR and spontaneous MT is associated with improved survival in G2 Astro MT patients, whereas prolongation of time to MT by treatment in G2 Oligo is not accompanied by a clear survival benefit. We provide a summary of median OS across all groups, as well as predictors of time to MT and OS in patients with MT (Figure 2).

## Discussion

In our retrospective clinical study of 193 *IDH* mutant glioma patients, we expand the understanding of MT, which denotes disease progression to a higher grade. MT is often defined based on imaging, but a previous study found that in progressive low-grade glioma patients, novel MRI enhancement and pathologic grade were discordant in more than 20% of cases.^17^ Compared with prior reports that rely upon imaging and may include misleading cases of nontransforming growth, our study exercises a stricter approach by defining MT based entirely on the pathology of resected tissue at subsequent surgeries. Furthermore, many studies on MT include patients of mixed *IDH* status, despite their distinct clinical courses and evidence that MT occurs more commonly among *IDH* mutant gliomas.^5^

We observed that compared with initial G2 Astro patients, initial G2 Oligo patients had a longer time to MT. Among initial G2 Astro patients, MT to G4 Astro (whether directly or stepwise) predicted worse OS than MT to G3 Astro. For G2 Astro patients, our data suggest that in the case of stepwise (G2 to G3 to G4 Astro) MT, MT from G3 to G4 Astro is likely to occur within a year following G3 Astro diagnosis. We found that MT was associated with poorer outcome among *IDH* mutant glioma patients initially diagnosed with G2 Astro, but not G3 Astro or G2 Oligo. A potential explanation for MT and non-MT G3 Astro patients performing similarly may have to do with the already aggressive, high-grade nature of G3 Astro. Moreover, MT from G2 Oligo to G3 Oligo may not have a negative impact due to the relatively indolent nature of oligodendrogliomas compared with astrocytomas.^2^ Although additional studies comparing larger subcohorts are required to validate the subtype-specific implications of MT, this finding provides preliminary evidence that efforts to mitigate MT risk are especially critical in patients initially diagnosed with G2 Astro.

In terms of clinical risk variables, we found that among G2 Astro MT patients, initial GTR is associated with prolonged MT-free survival and OS compared with STR or biopsy. Previous studies have also linked lower extent of resection to decreased progression-free survival.^9^ Additionally, one study indicates that *IDH* mutant G2 Astro patients benefit the most from greater extent of resection.^10^ In conjunction with these findings, our data suggest that maximizing extent of resection at initial surgery is an especially robust strategy for reducing MT risk in the case of G2 Astro. Interestingly, supratotal resection has also been associated with prolonged progression-free survival as well as OS in *IDH* mutant low-grade glioma patients,^11^ though further analyses are necessary to determine whether there is a significant advantage of supratotal resection over GTR.

We also found that treatment with chemotherapy and/or radiation delays MT in G2 Oligo patients. This aligns with existing literature that radiotherapy is effective at prolonging MT-free survival,^19^ wheres chemoradiation has a similar beneficial effect in G2 Oligo but may contribute to accelerated MT in G2 Astro.^18^ However, our data further suggests that spontaneous MT correlates with better outcome. This difference may be due to selection bias, where patients with aggressive-presenting tumors are more likely to receive treatment. Still, previous literature has implicated TMZ and its hypermutating effects as a contributing factor to MT.^8,12,13^ Given this, further analyses are required to understand the nuanced, subtype-specific role of treatment in relation to MT and survival.

Closer analysis of the 34 spontaneous MT patients provided an opportunity to examine the natural history of MT, independent of the effects of standard-of-care treatment. Since G2 Oligo did not exhibit later spontaneous MT compared with G2 Astro, the time to MT difference observed between all G2 Oligo and G2 Astro patients may be attributed to the overriding effects of treatment. Older age predicted earlier spontaneous MT, whereas GTR predicted later spontaneous MT, which aligns with another study of 92 *IDH* mutant low-grade glioma patients who received observation only.^20^ Interestingly, we also found that development of CEnew was less likely to precede spontaneous MT as opposed to treated MT, though further studies should be done to investigate the contribution of treatment effect. A caveat is that while most spontaneous MT patients (*n* = 30) were under watchful observation only prior to MT, 4 were treated with small-molecule inhibitors. The impact of these nonstandard-of-care treatments is uncertain, so additional studies should be done to evaluate true biological changes that accompany spontaneous MT.

By analyzing the triggers for patients’ additional surgeries in our cohort, we were also able to retrospectively assess the accuracy of CEnew in determining pathologically confirmed MT. Sixty-five percent of second surgeries triggered by CEnew resulted in MT, but 33% of surgeries for reasons unrelated to CEnew also resulted in MT. We calculated an overall positive predictivity of 65%, negativity predictivity of 67%, sensitivity of 75%, and specificity of 55%, which were comparable to the trend in values reported by a previous study (82%, 77%, 92%, and 57%, respectively).^17^ These findings indicate that CEnew is an imperfect indicator of MT, and the absence of CEnew does not necessarily eliminate the possibility of MT. Since a clinician’s response to CEnew often depends on a tumor’s initial presentation, we also evaluated the predictive value of preoperative enhancement and found no significant correlation to MT incidence by Fisher’s exact test (Supplementary Table S10).

With the exception of 2 PsP patients, 65 of the non-MT patients (34% of cohort) experienced pathologically determined nontransforming progression. By subtype, nontransforming progression occurred in 20 (24%) G2 Astro, 23 (49%) G3 Astro, and 22 (36%) G2 Oligo patients. The most common trigger for second surgery was T2 change (*n* = 30, 46%), followed by CEnew (*n* = 25, 38%) and lastly, redo craniotomy (*n* = 10, 15%). Given the considerable overlap in surgical triggers between groups, our study demonstrates the challenge in distinguishing MT from nontransforming progression without pathological confirmation. This displays the importance of the ongoing search for additional markers for MT, such as specific MRI parameters^14^ or genomic shifts in tumor biology.^15^

Although relying on pathology to define MT reduces the possibility of false calls of MT, this methodology introduces limitations to our study. In our cohort of 126 MT patients, 41 (33%) had at least 1 radiographic progression that did not lead to surgery prior to their documented instance of MT (Supplementary Table S11). In the interval between initial surgery and first radiographic progression, 9 (22%) of these patients received radiation, 7 (17%) received chemotherapy, 14 (34%) received chemoradiation, and 11 (27%) did not receive prior treatment. Because our definition of MT required surgical confirmation, our study may not have been an accurate representation of clinical course for these patients. Only patients who were offered and opted for surgery were included, which fails to account for patients that experienced MT but did not undergo surgery for any reason. To verify that this did not artificially select for patients with worse outcomes, we compared the OS of our cohort and other *IDH* mutant lower-grade glioma patients with initial surgery only (Supplementary Figure S6) and found no differences in any subtype. Considering the ongoing monitoring of patients in our partially censored cohort, it is possible that patients in the non-MT group will later exhibit MT. Since the reported incidence rates of MT are dependent on the extent of follow-up and therefore can be considered estimations only, we also analyzed time to MT to evaluate the propensity for MT across different glioma subtypes. Another caveat is that no central review of MRI or pathology was performed. Moreover, our multivariate analyses yielded unexpected correlations, suggesting that male sex and lower KPS predict better OS in G2 Astro MT patients. Because these covariates have been known to predict worse outcome,^21^ we do not believe these findings to be reflective of true biological differences, but rather these characteristics being skewed in our subcohort of G2 Astro MT patients.

Overall, our results support a growing body of evidence that MT alters the prognosis of glioma patients. We show that the timing and survival effect of MT vary across glioma subtypes and that adjusting clinical response, specifically extent of resection and treatment, can slow down the onset of MT. Given the expanding role of molecular findings in glioma diagnosis and treatment, our study reveals the relevance of histopathological tumor grade and molecular subtype, both of which translate to clinical consequences. Our findings also emphasize the importance of searching for novel strategies to delay MT, through contemporary efforts such as metabolic reprogramming,^22^ epigenetic targeting,^23^ and *IDH* inhibitors.^24^ While additional studies should be conducted to investigate the molecular underpinnings of MT, this study allows for an enhanced understanding of this phenomenon from a clinical perspective and holds important insights for the diagnosis and treatment of *IDH* mutant lower-grade glioma patients.

## Supplementary Material

vdad036_suppl_Supplementary_Material
