# Supplementary material for: A single-institution retrospective analysis of pathologically determined malignant transformation in IDH mutant glioma patients
Source: Neurooncol Adv. 2023 Apr 11;5(1):vdad036. doi: 10.1093/noajnl/vdad036 (PMC10162112; doi:10.1093/noajnl/vdad036)

**Supplemental Figure 1.** Flowchart depicting the patient selection process.


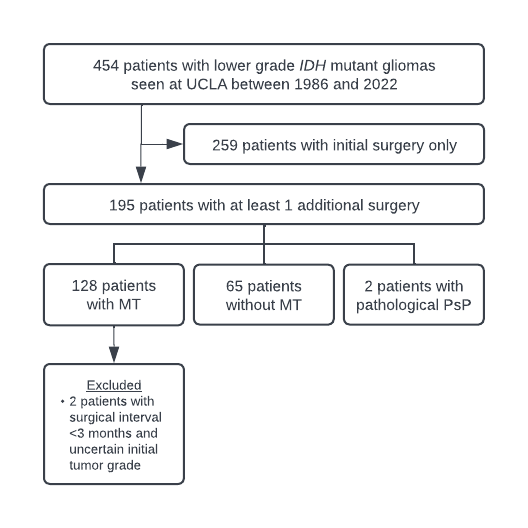


**Supplemental Table 1.** Table documenting the additional surgery and extent of resection (EOR) when MT was observed. For the 10 patients that experienced stepwise MT from G2 to G3 to G4 Astro, numbers with (*) refer to the intermediate G3 Astro surgery while numbers without asterisks indicate the G4 Astro surgery.

| MT surgery and EOR | G2 to G3 Astro (n = 26) | G2 to G4 Astro (n = 28) | G2 to G3 to G4 Astro (n = 10) | G3 to G4 Astro (n =23) | G2 to G3 Oligo (n = 39) |
| --- | --- | --- | --- | --- | --- |
| 2nd surgery | 23 | 23 | 9 *, 0 | 21 | 26 |
| STR or biopsy | 10 | 14 | 4 *, 0 | 11 | 14 |
| GTR | 13 | 7 | 5 *, 0 | 8 | 11 |
| Unknown | 0 | 2 | 0 *, 0 | 2 | 1 |
| 3rd surgery | 2 | 3 | 1 *, 8 | 2 | 11 |
| STR or biopsy | 0 | 2 | 1 *, 5 | 0 | 4 |
| GTR | 2 | 1 | 0 *, 3 | 2 | 5 |
| Unknown | 0 | 0 | 0 *, 0 | 0 | 2 |
| 4th+ surgery | 1 | 2 | 0 *, 2 | 0 | 2 |
| STR or biopsy | 1 | 0 | 0 *, 2 | 0 | 1 |
| GTR | 0 | 2 | 0 *, 0 | 0 | 1 |
| Unknown | 0 | 0 | 0 *, 0 | 0 | 0 |

**Supplemental Table 2.** Table summarizing the triggers for 2^nd^ surgery of all patients who received additional surgeries (n = 193).

| Initial diagnosis | CEnew | T2 change | Repeat craniotomy |
| --- | --- | --- | --- |
| G2 Astro | 51 | 27 | 7 |
| G3 Astro | 37 | 5 | 5 |
| G2 Oligo | 30 | 27 | 4 |
| All | 118 | 59 | 16 |

**Supplemental Figure 2.** Flowchart depicting all outcomes, beginning with initial diagnosis. Median OS and median times to MT, above respective arrows, are shown in months.


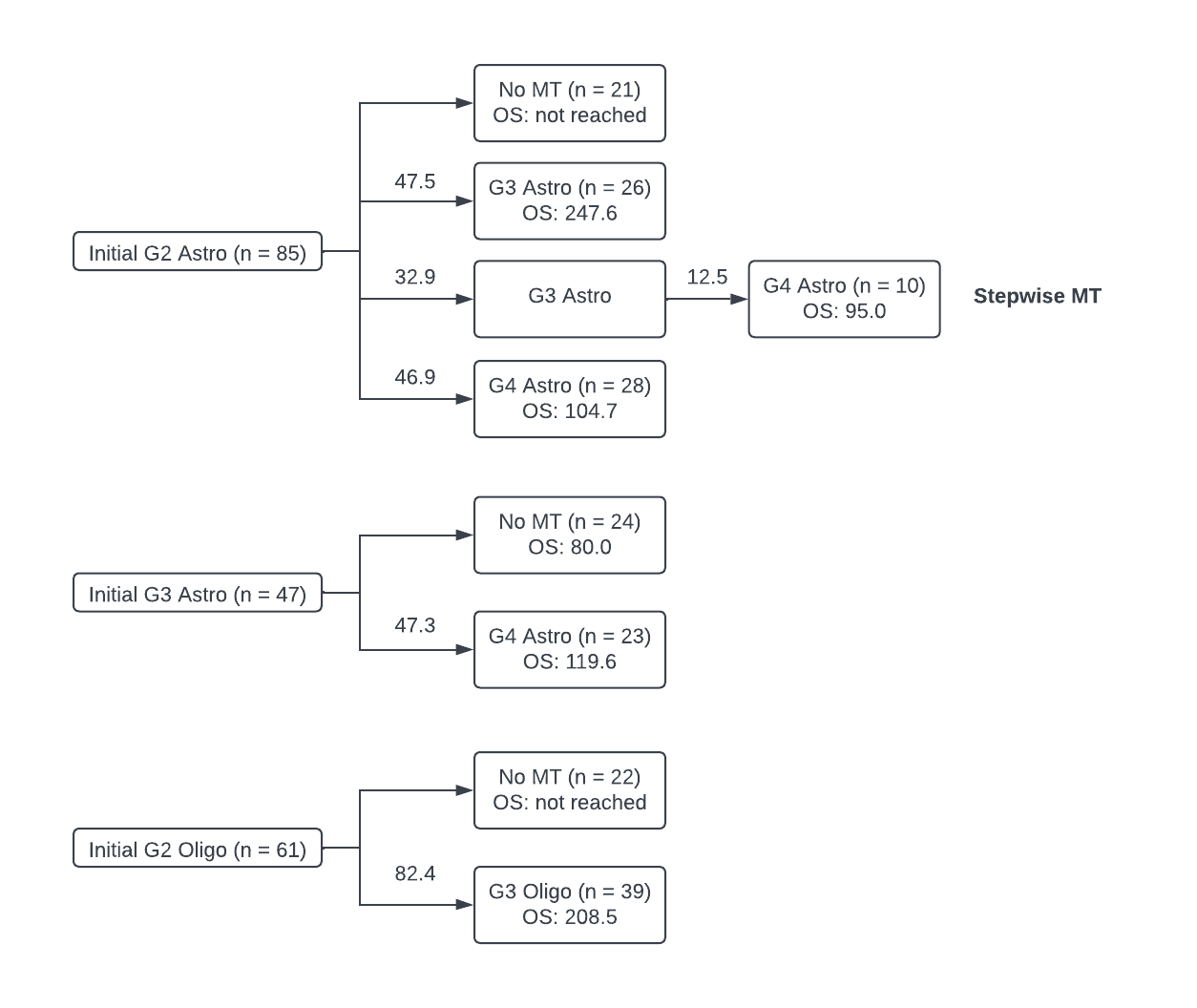


**Supplemental Figure 3.** Univariate Kaplan Meier analysis comparing (A) time to MT from G2 to G3 Astro of stepwise MT patients vs. initial G2 to G3 Astro only patients, (B) time to MT from G3 to G4 Astro of stepwise MT patients vs. initial G3 to G4 Astro patients, and (C) time to MT from G2 to G4 Astro of stepwise MT patients vs. G2 to G4 Astro patients who did not receive an intermediate diagnosis of G3 Astro.


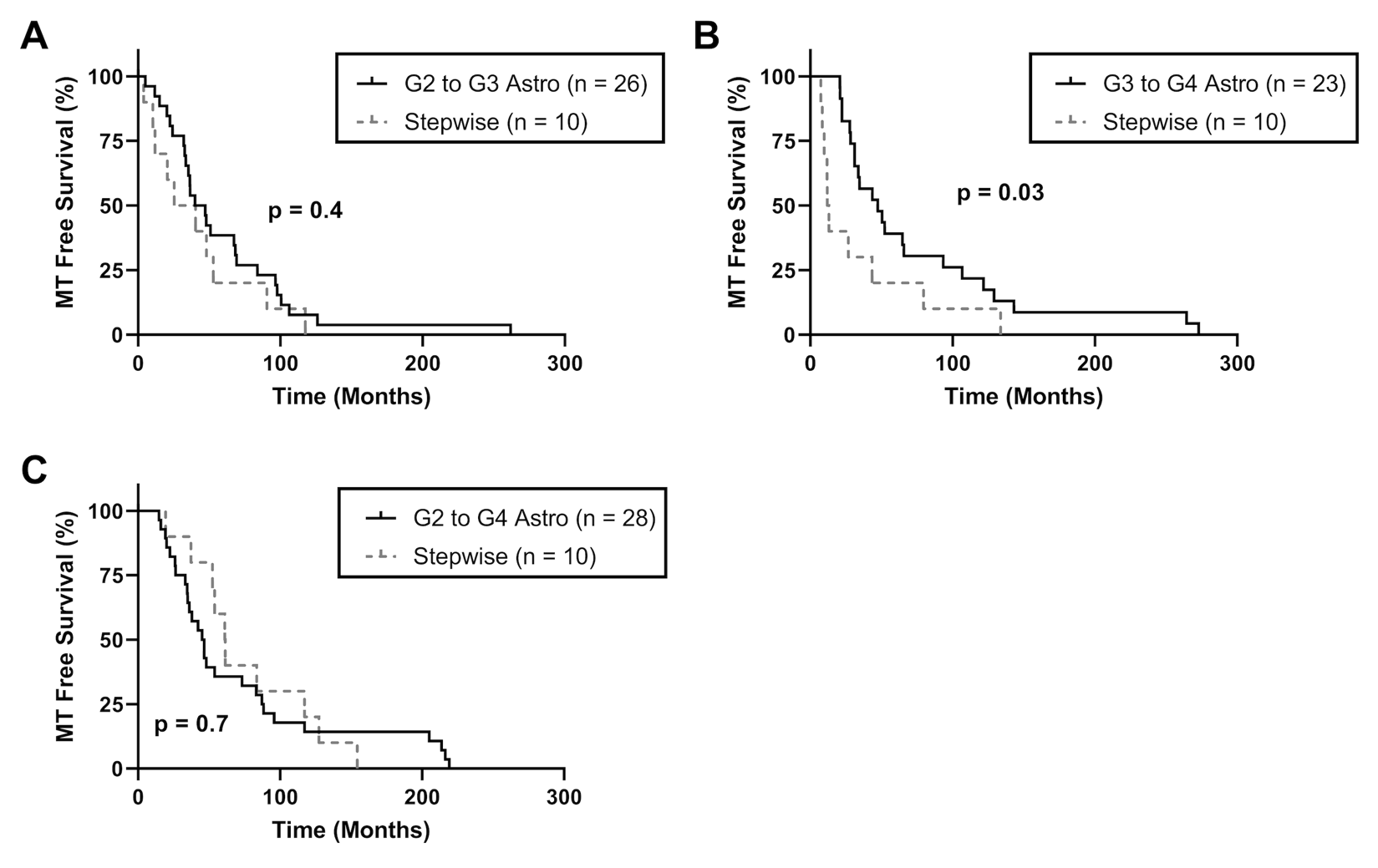


**Supplemental Table 3.** Multivariate analysis of OS and time to MT in patients that experience MT from G2 to G4 Astro. The Stepwise G2 to G3 to G4 Astro covariate is added to examine the prognostic impact of stepwise MT compared to direct MT from G2 to G4 Astro.

|  | OS of LA to G4 Astro (n = 37) | | | Time to MT of G2 to G4 Astro (n = 37) | | |
| --- | --- | --- | --- | --- | --- | --- |
|  | HR | p value | 95% CI | HR | p value | 95% CI |
| Age at initial surgery | 1.01 | 0.6 | [0.97, 1.06] | 1.05 | 0.03* | [1.00, 1.09] |
| Male (ref. female) | 0.53 | 0.1 | [0.24, 1.18] | 0.35 | 0.02* | [0.15, 0.82] |
| KPS ≤ 70 | 0.08 | 0.01* | [0.01, 0.56] | 0.15 | 0.01* | [0.03, 0.65] |
| GTR (ref. STR/biopsy) | 0.31 | 0.02* | [0.12, 0.83] | 0.62 | 0.2 | [0.27, 1.38] |
| Stepwise G2 to G3 to G4 Astro (ref. G2 to G4 Astro directly) | 1.16 | 0.8 | [0.47, 2.83] | 1.97 | 0.1 | [0.88, 4.43] |
| Events (%) | 28 (76) | | | 37 (100) | | |

**Supplemental Table 4.** Table of positive predictivity, negative predictivity, sensitivity, and specificity values of CEnew as a marker of MT across different glioma subtypes.

| Initial diagnosis | Positive predictivity (%) | Negative predictivity (%) | Sensitivity (%) | Specificity (%) |
| --- | --- | --- | --- | --- |
| G2 Astro | 76 | 53 | 71 | 60 |
| G3 Astro | 54 | 90 | 95 | 35 |
| G2 Oligo | 60 | 74 | 69 | 66 |
| All | 65 | 67 | 75 | 55 |

**Supplemental Figure 4.** Univariate Kaplan Meier analysis comparing OS and time to MT of patients that received initial subtotal resection(STR)/biopsy vs. gross total resection (GTR) for (A) all (OS: p = 0.07, time to MT: p = 0.4), (B) initial G2 Astro (OS: p = 0.03, time to MT: p = 0.03), (C) initial G3 Astro (OS: p = 0.9, time to MT: p = 0.6), and (D) initial G2 Oligo (OS: p = 0.8, time to MT: p = 0.1).


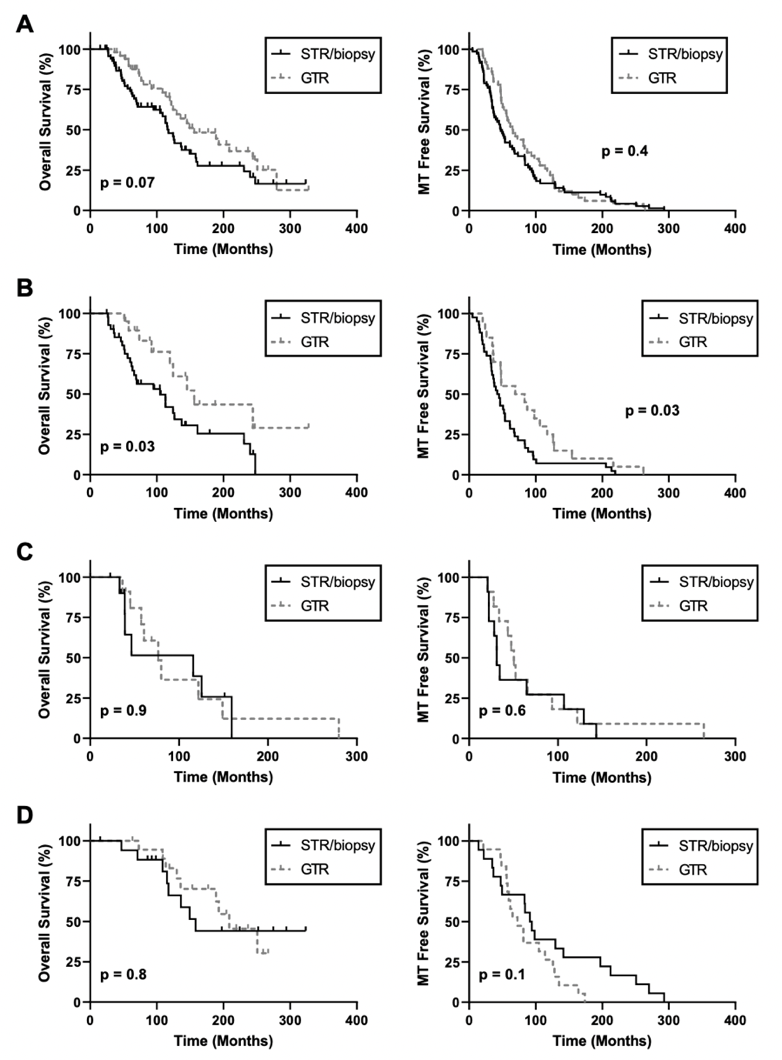


**Supplemental Figure 5.** Univariate Kaplan Meier analysis comparing (A) OS (p = 0.01), (B) resOS (p < 0.0001), and (C) time to MT (p = 0.01) of patients that underwent treated MT vs. spontaneous MT.


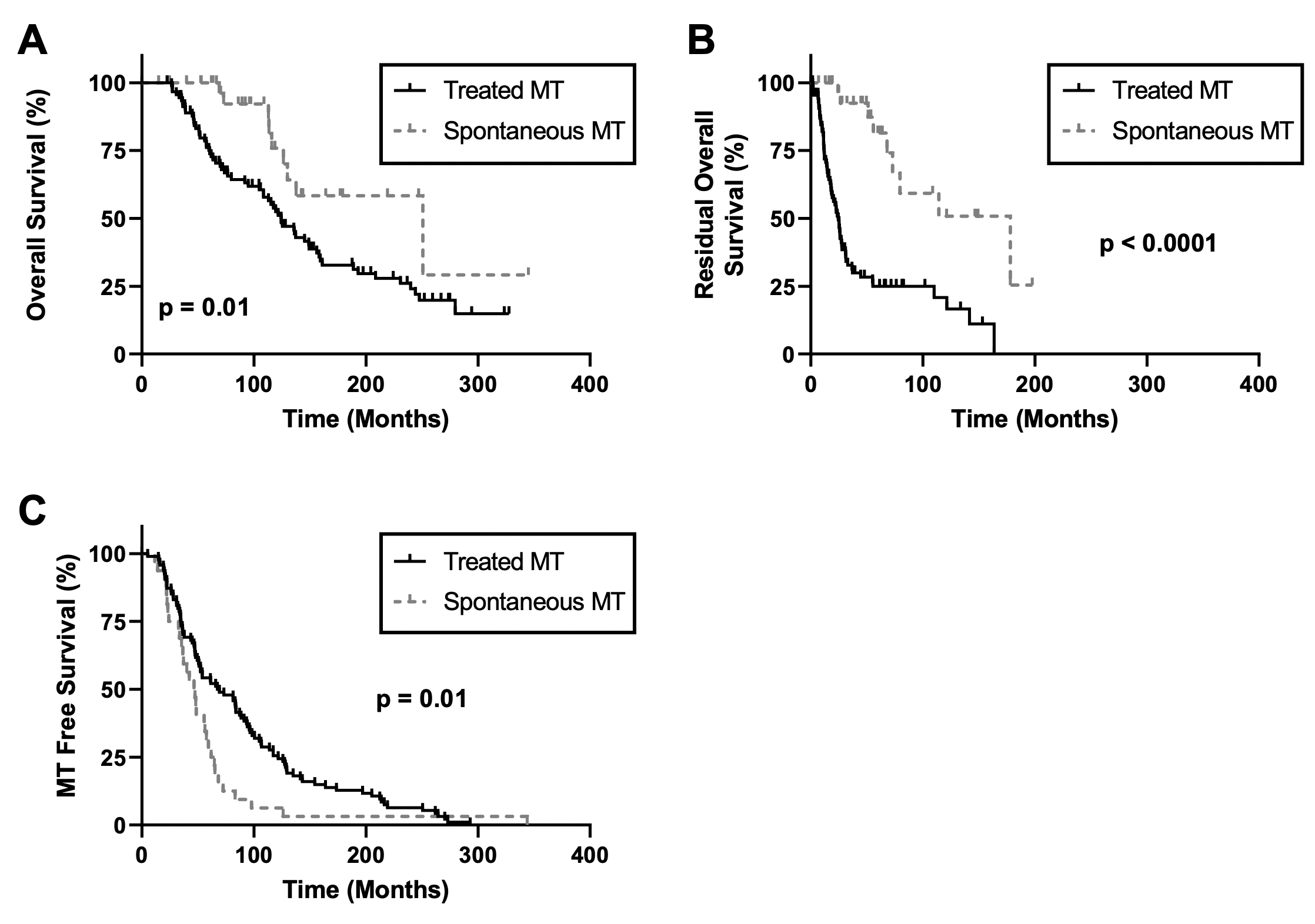


**Supplemental Table 5.** Multivariate analysis of time to spontaneous MT in untreated MT patients with known EOR.

| Variable (Time to MT) | All sMT (n = 34) | | |
| --- | --- | --- | --- |
|  | HR | p value | 95% CI |
| Age at initial surgery | 1.07 | 0.001* | [1.03, 1.11] |
| Male (ref. female) | 0.76 | 0.5 | [0.35, 1.65] |
| KPS ≤ 70 | 0.11 | 0.3 | [0.01, 4.61] |
| GTR (ref. STR/biopsy) | 0.35 | 0.02* | [0.14, 0.87] |
| Initial G3 Astro (ref. G2 Astro) | 4.1 | 0.5 | [0.10, 16.98] |
| Initial G2 Oligo (ref. G2 Astro) | 1.48 | 0.4 | [0.58, 3.79] |

**Supplemental Table 6.** Table comparing characteristics of treated MT vs. spontaneous MT patients. Due to patients with unavailable *MGMT* methylation (n = 50), pre-operative enhancement (n = 62), initial extent of resection (n = 5), and initial pathology descriptions (n = 53), percentages of patients with known statuses are shown for these characteristics. (*) indicates a significant association by Fisher’s exact test.

| Characteristics | Treated MT | Spontaneous MT | Fisher's exact test |
| --- | --- | --- | --- |
| N | 92 | 34 | - |
| Median age at initial surgery | 32.3 | 36.2 | - |
| Male (%) | 56 (61) | 20 (35) | p = 0.8 |
| *MGMT* methylated (% of known) | 36 (67) | 12 (55) | p = 0.4 |
| Pre-operative enhancing (% of known) | 19 (40) | 8 (47) | p = 0.8 |
| Initial gross total resection (% of known) | 32 (36) | 18 (58) * | p = 0.03 |
| Concerning initial tumor pathology (% of known) | 29 (58) | 13 (57) | p = 1 |
| CEnew as trigger for MT surgery (%) | 78 (85) * | 22 (65) | p = 0.02 |

**Supplemental Table 7.** Multivariate analysis of OS in all patients in cohort with known EOR, stratified by initial diagnoses. MT covariate is included to examine prognostic impact of MT.

| Variable (OS) | All (n = 186) | | | G2 Astro (n = 82) | | | G3 Astro (n = 45) | | | G2 Oligo (n = 59) | | |
| --- | --- | --- | --- | --- | --- | --- | --- | --- | --- | --- | --- | --- |
|  | HR | p value | 95% CI | HR | p value | 95% CI | HR | p value | 95% CI | HR | p value | 95% CI |
| Age at initial surgery | 1.02 | 0.1 | [0.99, 1.04] | 1.00 | 1 | [0.97, 1.03] | 1.06 | 0.04* | [1.00, 1.11] | 1.04 | 0.1 | [0.99, 1.09] |
| Male (ref. female) | 0.83 | 0.4 | [0.52, 1.32] | 0.59 | 0.1 | [0.30, 1.18] | 1.10 | 0.8 | [0.43, 2.77] | 0.84 | 0.7 | [0.33, 2.18] |
| KPS ≤ 70 | 1.07 | 0.9 | [0.50, 2.26] | 0.38 | 0.1 | [0.10, 1.40] | 2.24 | 0.3 | [0.55, 9.12] | 3.87 | 0.04* | [1.03, 14.48] |
| GTR (ref. STR/biopsy) | 0.78 | 0.3 | [0.49, 1.24] | 0.41 | 0.02* | [0.19, 0.88] | 1.77 | 0.2 | [0.72, 4.38] | 1.07 | 0.9 | [0.42, 2.75] |
| Initial G3 Astro (ref. G2 Astro) | 1.92 | 0.01* | [1.14, 3.22] | - | - | - | - | - | - | - | - | - |
| Initial G2 Oligo (ref. G2 Astro) | 0.44 | 0.004* | [0.25, 0.78] | - | - | - | - | - | - | - | - | - |
| MT (ref. no MT) | 2.54 | 0.0009* | [1.46, 4.42] | 4.26 | 0.02* | [1.28, 14.15] | 1.54 | 0.4 | [0.60, 3.51] | 2.62 | 0.1 | [0.76, 8.97] |
| Events (%) | 87 (47) | | | 40 (49) | | | 26 (58) | | | 21 (36) | | |

**Supplemental Table 8.** Table comparing characteristics of G2 to G3 Astro only vs. stepwise G2 to G3 to G4 Astro patients. Due to patients with unavailable *MGMT* methylation (n = 15), pre-operative enhancement (n = 18), initial extent of resection (n = 1), initial pathology descriptions (n = 17), and CEnew before/after G3 Astro surgery (n = 6), percentages of patients with known statuses are shown for these characteristics.

| Characteristics | G2 to G3 Astro only | G2 to G3 to G4 Astro | Fisher's exact test |
| --- | --- | --- | --- |
| N | 26 | 10 | - |
| Median age at initial surgery | 31.4 | 34.8 | - |
| Male (%) | 16 (64) | 7 (70) | p = 0.7 |
| *MGMT* methylated (% of known) | 8 (53) | 4 (67) | p = 0.7 |
| Pre-operative enhancing (% of known) | 4 (33) | 2 (33) | p = 1 |
| Initial gross total resection (% of known) | 10 (40) | 3 (30) | p = 0.7 |
| Chemotherapy and/or radiation prior to G3 Astro surgery (%) | 14 (54) | 7 (70) | p = 0.5 |
| Concerning initial pathology (% of known) | 6 (43) | 4 (67) | p = 0.6 |
| CEnew prior to G3 Astro surgery (% of known) | 5 (25) | 5 (50) | p = 0.2 |
| GTR at G3 Astro surgery (%) | 15 (58) | 5 (50) | p = 0.7 |
| Chemotherapy and/or radiation after G3 Astro surgery (%) | 25 (96) | 9 (90) | p = 0.5 |

**Supplemental Table 9.** Multivariate analysis of residual overall survival (resOS) in MT patients with known EOR, stratified by initial diagnoses.

| Variable (resOS) | All (n = 121) | | | G2 Astro (n = 62) | | | G3 Astro (n = 22) | | | G2 Oligo (n = 37) | | |
| --- | --- | --- | --- | --- | --- | --- | --- | --- | --- | --- | --- | --- |
|  | HR | p value | 95% CI | HR | p value | 95% CI | HR | p value | 95% CI | HR | p value | 95% CI |
| Age at initial surgery | 1.01 | 0.7 | [0.98, 1.03] | 1.00 | 0.8 | [0.96, 1.03] | 1.03 | 0.4 | [0.97, 1.09] | 1.05 | 0.2 | [0.97, 1.14] |
| Male (ref. female) | 1.11 | 0.7 | [0.66, 1.87] | 0.71 | 0.3 | [0.35, 1.44] | 4.56 | 0.04* | [1.05, 18.91] | 1.2 | 0.8 | [0.34, 4.19] |
| KPS ≤ 70 | 2.01 | 0.09 | [0.89, 4.54] | 0.67 | 0.6 | [0.15, 3.07] | 7.85 | 0.02* | [1.35, 45.47] | 6.41 | 0.01* | [1.53, 26.85] |
| GTR (ref. STR/biopsy) | 0.57 | 0.04* | [0.33, 0.97] | 0.47 | 0.06 | [0.22, 1.03] | 0.92 | 0.9 | [0.25, 3.34] | 0.57 | 0.4 | [0.17, 1.93] |
| Initial G3 Astro (ref. G2 Astro) | 3.44 | 0.0002* | [1.79, 6.62] | - | - | - | - | - | - | - | - | - |
| Initial G2 Oligo (ref. G2 Astro) | 0.59 | 0.1 | [0.32, 1.10] | - | - | - | - | - | - | - | - | - |
| No treatment (ref. chemotherapy and/or radiation) | 0.20 | <0.0001* | [0.10, 0.42] | 0.18 | 0.0007* | [0.07, 0.49] | 0.08 | 0.06 | [0.01, 1.09] | 0.3 | 0.1 | [0.07, 1.29] |
| Events (%) | 70 (58) | | | 37 (60) | | | 16 (73) | | | 17 (46) | | |

**Supplemental Table 10.** Table evaluating the predictive potential of pre-operative enhancement with regards to MT outcome in all, G2 Astro, G3 Astro, and G2 Oligo patients with available MRI data.

| Initial diagnosis | Pre-operative enhancement | Total | MT (%) | No MT (%) | Fisher's exact test |
| --- | --- | --- | --- | --- | --- |
| G2 Astro | Enhancing | 12 | 10 (83) | 2 (17) | p = 0.3 |
|  | Nonenhancing | 33 | 21 (64) | 12 (36) |  |
| G3 Astro | Enhancing | 12 | 7 (58) | 5 (42) | p = 0.7 |
|  | Nonenhancing | 16 | 8 (50) | 8 (50) |  |
| G2 Oligo | Enhancing | 11 | 8 (73) | 3 (27) | p = 0.7 |
|  | Nonenhancing | 20 | 12 (60) | 8 (40) |  |
| All | Enhancing | 35 | 25 (71) | 10 (29) | p = 0.3 |
|  | Nonenhancing | 69 | 41 (59) | 28 (41) |  |

**Supplemental Table 11.** Table documenting the number of radiographic progressions without surgery prior to MT in all, G2 Astro, G3 Astro, and G3 Oligo MT patients.

| Initial diagnosis | 0 non-surgical progressions prior to MT | 1 non-surgical progression prior to MT | 2+ non-surgical progressions prior to MT | Median non-surgical progressions prior to MT |
| --- | --- | --- | --- | --- |
| G2 Astro | 47 | 11 | 6 | 0 |
| G3 Astro | 15 | 5 | 3 | 0 |
| G2 Oligo | 23 | 10 | 6 | 0 |
| All | 85 | 26 | 15 | 0 |

**Supplemental Figure 6.** Univariate Kaplan Meier analysis comparing OS of patients that received initial surgery only vs. additional surgeries for (A) all relevant glioma subtypes (p = 0.2), (B) initial G2 Astro (p = 0.8), (C) initial G3 Astro (p = 0.1), (D) initial G2 Oligo (p = 0.7).


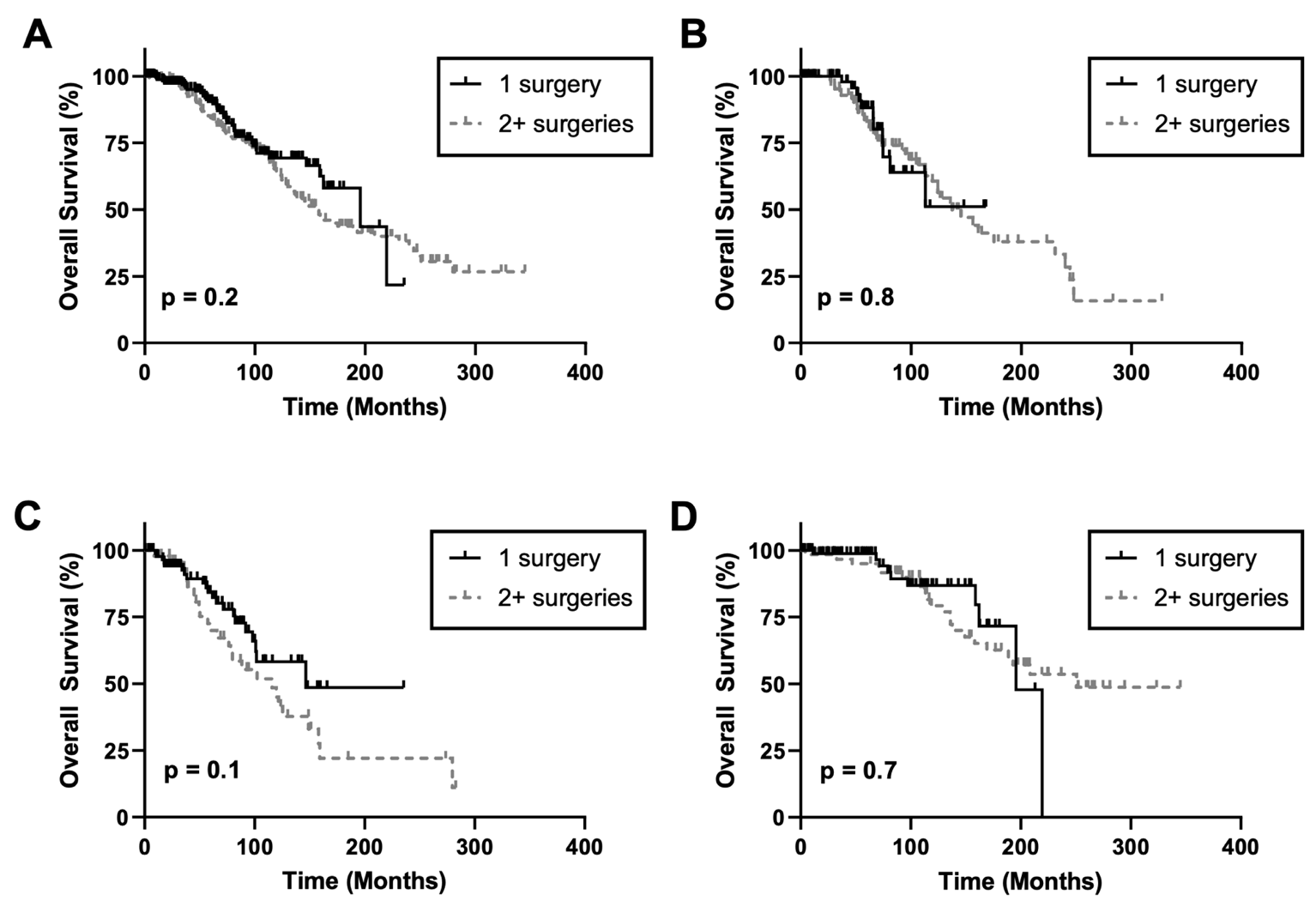

Supplement: vdad036_suppl_Supplementary_Material [file vdad036_suppl_supplementary_material.docx]
